# Caputo–Fabrizio fractional model of MHD second grade fluid with Newtonian heating and heat generation

**DOI:** 10.1038/s41598-022-26080-7

**Published:** 2022-12-26

**Authors:** Sehra, Wajeeha Iftikhar, Sami Ul Haq, Saeed Ullah Jan, Ilyas Khan, Abdullah Mohamed

**Affiliations:** 1 Shaheed Benazir Bhutto Women University Peshawar, Peshawar, 25000 Khyber Pakhtunkhwa Pakistan; 2 Department of Mathematics, Islamia College Peshawar, Peshawar, 25000 Khyber Pakhtunkhwa Pakistan; 3 Department of Mathematics, College of Science in Zulfi, Majmaah University, Al-Majmaah, 11952 Saudi Arabia; 4 Research Centre, Future University in Egypt, New Cairo, 11835 Egypt

**Keywords:** Mathematics and computing, Physics

## Abstract

In this research article the heat transfer of generalized second grade fluid is investigated with heat generation. The fluid flow is analyzed under the effects of Magneto hydrodynamics over an infinite vertical flat plate. The Newtonian heating phenomenon has been adopted at the boundary. For this purpose the problem is divided into two compartments i.e. momentum equation and energy equations. Some specific dimensionless parameters are defined to convert the model equations into dimensionless system of equations. The solutions for dimensionless energy and momentum equations are obtained by using the Laplace transform technique. From obtained results by neglecting magneto hydrodynamic effects and heat source some special case are achieved which are already published in literature. The case for which the fractional parameter approaches to the classical order is also discussed and it has been observed that it is convergent. Finally, the influences of different physical parameters are sketched graphically. It has been observed that for increasing values of Prandtl number the velocity and temperature decreases, for increasing values of Grashof number the velocity of the fluid increases. Also it has been investigated that for increasing values of fractional parameter the velocity and temperature of the fluid increases.

## Introduction

Basically the non-Newtonian fluids are divided into three main groups according to their actions with shear stress i.e. integral type, differential type and rate-type. (1) The integral type fluids are those fluids, whose shear stress is hardly dependent upon the shear rate. (2) the fluids whose shear strain and shear rate are related to each other are called differential type. (3) those fluids which have the properties of viscosity and elasticity are known as rate type. Grade Second fluids belongs to differential type which is more famous among the various popular models of the non-Newtonian fluids. The pioneers who designed the second grade fluid model were Coleman and Noll^1^. Subsequently, this framework was used for the analysis of different problems whose construction is relatively simple. The second-order Rivlin–Erickson equations has been applied to explain the pattern of a non-Newtonian fluid flowing unsteadily upon a flat surface as like the Couette flow and Poiseuille flux^2^. Many authors have been studied some of the unsteady flows of the second grade fluid^3,4^. Derivatives are mostly used to formulate the real world problems into mathematical models. In particular, fractional derivatives are more suitable for some well-known problems than regular derivative. In recent past the application of fractional order derivatives has been expanded in distinct fields. Especially, in dynamics of fluid, viscoelasticity, bioengineering, electrochemistry, finance, fluent currents tracers and in signal processing. Particularly, fractional derivatives are more suitable for some major problems than regular derivatives^5–7^. The fractional derivative models are used widely in different directions like, polymers for glass transition and glass state referable that the fractional derivative models can easily explains the complex behavior of a viscoelastic fluid^8–11^. Free convection flow of generalized viscous fluid upon a vertical plate with chemical and Newtonian heating is investigated in^12^. The fractional second grade fluid investigated by using the Caputo fractional derivatives^13^. In the near past Caputo and Fabrizio have presented a new fractional operator know as Caputo–Fabrizio operator, which has been used in many theoretical real word phenomena^14^. The generalized second grade fluid is investigated with Caputo–Fabrizio differential operation by adopting the Laplace transformation technique, and obtained the exact solutions to the problem^15^. Exact analytical for viscous fluid with non-singular kernal differential operator is gained^16^. Due to the rising concern of fractional derivative modeling many fractional models have been modeled using the existing models of fluid^17–19^. The convection heat-mass transfer of generalized grade second fluid is analyzed, and results achieved by Caputo–Fabrizio is compared Atangana Baleanu fractional operator^20^. The author analyzed heat transfer in convective flow of second grade fluid subjected to Newtonian heating using Atangana Baleanu fractional and Caputo Fabrizio fractional derivative. They also carried out the comparison of the two approaches^21^. The heat transfer in MHD flow of generalized second grade fluid with porosity in the medium by adopting Caputo Fabrizio derivative^22^. Heat transfer during the unsteady magneto hydrodynamic flow of a differential-type fluid in Forchhiemer medium was analyzed numerically^23^. The unsteady magneto hydrodynamic flow of viscoelastic fluid flowing in a porous medium^24^. Heat transfer during the incompressible time-dependent flow of Maxwell viscoelastic fluid by some stretching surface with chemical reaction and radiation source was investigated in^25^. The analysis of rate type anomalous Nano-fluid with Caputo non-integer order derivative flowing unsteadily was studied in^26^. The two-dimensional and two-directional MHD flow of fractional viscoelastic fluid was analyzed in^27^. The authors studied the unequal diffusivities of chemical species in a Forchhiemer medium by using Scott Blair model of viscoelastic fluid with unsteady convection in^28^. The author studied the effects of mixed convection with thermal radiation and chemical by using the space–time coupled Cattaneo–Friedrich Maxwell Model with Caputo fractional derivatives in a porous medium^29^. The authors used the fractional calculus to analyzed the thermo-diffusion phenomenon numerically in a Darcy medium^30^. Khan and Rasheed studied the numerical implementation and error analysis with variable heat flux of coupled non-linear fractional viscoelastic fluid in^31^. Mumtaz et al.^32^ studied the computational simulation of viscoelastic model of Scott Blair to the hybrid fractional Nanofluid in a porous Darcy medium.

The main aim of this article is to extend the application of Caputo Fabrizio fractional derivative to the second grade fluid with magneto hydrodynamic effects in addition to the heat generation. Also the considered Newtonian heating is adopted at the boundary. The exact analytical solution has been achieved by using Laplace transformation on the dimensionless equations of the problem with suitable initial and boundary conditions. From obtained results by neglecting magneto hydrodynamic and heat source some special case are achieved which are published in literature. The case for which the fractional parameter approaches to the classical order also discussed and it has been observed that it is convergent. The exact solution of the problem is represented graphically to visualize the effects of physical parameters like time fractional, Magneto hydrodynamic, Prandtl number, Eta and Grashof Number etc.

## Mathematical analysis of the problem

**Figure 1:**
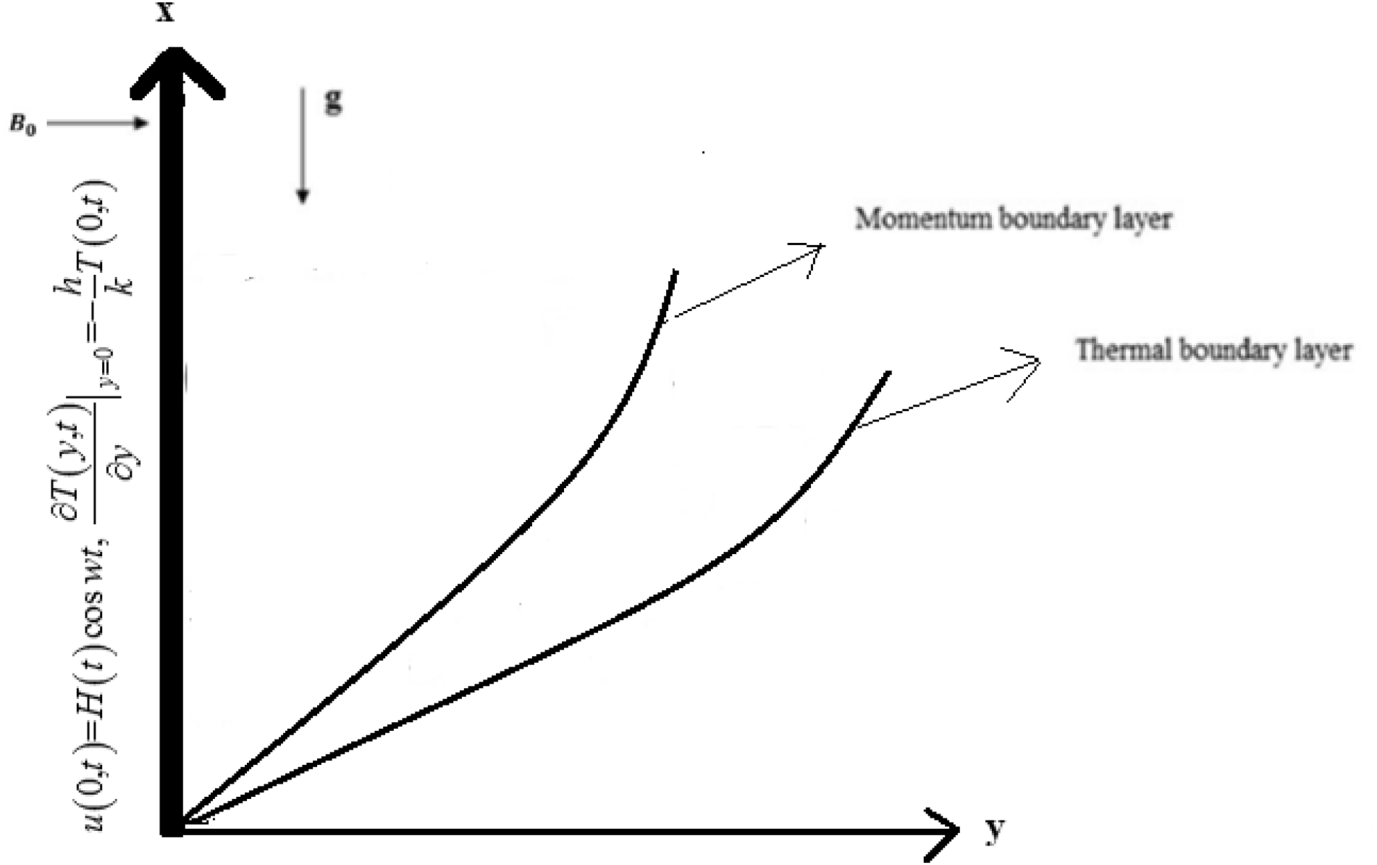
Geometry of the problem.

Consider the second grade fluid of unsteady flow over in an infinite upright plate with Newtonian heating at the boundary, flow direction is x-axis and y-axis is perpendicular to the flat plate. When $t{ = 0}$ both the fluid and the plate are at rest and the fluid temperature is $T_{\infty }$. But as time start i.e., for $t \, \succ {0}$ then temperature is ${\frac{\partial T\left(y,t\right)}{\partial y}|}_{y=0}=-\frac{h}{k}T(0,t)$ and fluid velocity becomes $u\left(0,t\right)=H(t)\mathrm{cos}\omega t$. The temperature and velocity are dependent on time t and y only. Now by usual Boussinesq’s approximation^16^. the unsteady flow is governed by the following set of partial differential equations. The flow of the fluid is represented by the following governing equations. The schematic diagram used in fluid flow problem is represented geometrically by Fig. 1.

1 $$\frac{\partial u\left(y,t\right)}{\partial t}=\nu \frac{{\partial }^{2}u\left(y,t\right)}{\partial {y}^{2}}+\frac{{\alpha }_{1}}{\rho }\frac{{\partial }^{3}u\left(y,t\right)}{\partial {y}^{2}\partial t}-\frac{\sigma {\beta }_{0}^{2}}{\rho }u\left(y,t\right)+g\beta \left(T\left(y,t\right)-{T}_{\infty }\right)$$

2 $$\rho {C}_{p}\frac{\partial T(y,t)}{\partial t}=k\frac{{\partial }^{2}T(y,t)}{\partial {y}^{2}}- Q\left(T\left(y,t\right)-{T}_{\infty }\right)$$

The initial (ICs) and boundary (BCs) conditions are

3 $$u \left(y,0\right)=0 , \quad T\left(y,0\right)={T}_{\infty } , \quad \forall y\ge 0$$

4 $$u\left(0,t\right)=H(t)\mathrm{cos}\omega t ,\quad {\frac{\partial T\left(y,t\right)}{\partial y}|}_{y=0}=-\frac{h}{k}T(0,t) ,\quad t>0$$

5 $$u\left(y,t\right) \to 0 ,\quad T\left(y,t\right) \to {T}_{\infty } , \quad as\; y \to \infty ,\quad t>0 \left[16\right].$$

where u denotes the fluid velocity [${\mathrm{ms}}^{-1}$], T denotes the fluid temperature [$\mathrm{k}]$,$\nu$ Kinematic viscosity $[ {\mathrm{m}}^{2}{\mathrm{s}}^{-1}]$, g Acceleration due to gravity [${\mathrm{ms}}^{-2}]$, $\rho$ Density of the fluid [${\mathrm{gkm}}^{-3}]$,$\sigma$ Electrical conductivity [S/m],${\beta }_{0}$ Uniform magnetic field, $\beta$ Volumetric coefficient of thermal expansion $[{\mathrm{K}}^{-1}]$,${C}_{p}$ Specifc heat at a constant pressure $[{\mathrm{jkg}}^{-1}{\mathrm{K}}^{-1}]$, k Termal conductivity of the fluid [${\mathrm{Wm}}^{-2}{\mathrm{K}}^{-1}]$, ${T}_{\infty }$ denotes Fluid Temperature far away from the sheet, Q denotes Heat generation,${\alpha }_{1}$ Second grade parameter.

## Dimensionless variables

The following dimensionless variables are utilized to gain a system of dimensionless governing equations from the set of dimensional governing equations.

6 $$\begin{aligned} y^{*} & = \frac{y}{\frac{k}{h}},\quad t^{*} = \frac{t}{{\frac{1}{\nu }\left( \frac{k}{h} \right)^{2} }},\quad u^{*} = \frac{u}{{\frac{g}{\nu }\left( \frac{k}{h} \right)^{2} }},\quad Pr = \frac{{\mu C_{p} }}{k},\quad T* = \frac{{T - T_{\infty } }}{{T_{\infty } }},\quad Gr = \beta T_{\infty } , \\ & \quad \gamma^{*} = \frac{{\alpha_{1} }}{\rho }\left( \frac{h}{k} \right)^{2} ,\quad \eta_{1} = \frac{{Qk^{2} }}{{\rho \nu C_{p} h^{2 } }},\quad M = \frac{{\sigma \beta_{0} }}{\rho \nu }\left( \frac{k}{h} \right)^{2} \\ \end{aligned}$$

Using these dimensionless variables given in Eq. (6) in Eqs. (1)–(2) and dropping out the star (*) notation, the governing Eqs. (1)–(2) take the simplest forms

7 $$\frac{\partial u(y,t)}{\partial t}=\frac{{\partial }^{2}u(y,t)}{\partial {y}^{2}}+\gamma \frac{{\partial }^{3}u(y,t)}{\partial {y}^{2}\partial t}-Mu\left(y,t\right)+GrT\left(y,t\right)$$

8 $$\frac{\partial T(y,t)}{\partial t}=\frac{1}{Pr}\frac{{\partial }^{2}T(y,t)}{\partial {y}^{2}}-{\eta }_{1}T\left(y,t\right)$$

To find a time-fractional order derivative model just interchange the time derivative of classical order with the time derivative of order $\alpha \in [\mathrm{0,1}]$, then as a result the following system of governing equations come into being:

9 $${D}_{t}^{\alpha }u\left(y,t\right)=\left(1+{D}_{t}^{\alpha }\right)\frac{{\partial }^{2}u(y,t)}{\partial {y}^{2}}-Mu\left(y,t\right)+GrT\left(y,t\right)$$

10 $${D}_{t}^{\alpha }T\left(y,t\right)=\frac{1}{Pr}\frac{{\partial }^{2}T(y,t)}{\partial {y}^{2}}-{\eta }_{1}T\left(y,t\right)$$

The appropriate non-dimensional initial and boundary conditions are

11 $$u \left(y,0\right)=0 , T\left(y,0\right)=0 , \forall y\ge 0$$

12 $$u\left(0,t\right)=H\left(t\right)\mathrm{cos}\omega t , {\frac{\partial T\left(y,t\right)}{\partial y}|}_{y=0}=-\left(T\left(0,t\right)+1\right) , t>0$$

13 $$u\left(y,t\right) \to 0 , T\left(y,t\right) \to {T}_{\infty } , as y \to \infty , t>0$$

The fractional operator used in this problem is Caputo–Fabrizio which is defined as under in (14) for $\alpha \in [\mathrm{0,1}]$,

14 $${D}_{t}^{\propto }u\left(y,t\right)=\frac{1}{1-\propto }{\int }_{0}^{t}exp\left(\frac{-\propto \left(t-\tau \right)}{1-\alpha }\right){u}^{^{\prime}}\left(\tau \right)d\tau$$

## Analytical Laplace transform solutions

In the above model, the solution of Eqs. (9) and (10) with initial and boundary conditions (11) and (12) will be obtained by applying Laplace transform.

### Solution for the temperature equation

With the help of Laplace technique we derived the differential equation

15 $$\frac{s\overline{T }(y,s)}{s\left(1-\alpha \right)+\alpha }=\frac{1}{Pr}\frac{{\partial }^{2}\overline{T }(y,s)}{\partial {y}^{2}}-{\eta }_{1}\overline{T }\left(y,s\right),$$

Let ${a}_{0}=\frac{1}{1-\alpha },$ then the equation

16 $$\frac{{a}_{0}s\overline{T }(y,s)}{s+\alpha {a}_{0}}=\frac{1}{Pr}\frac{{\partial }^{2}\overline{T }(y,s)}{\partial {y}^{2}}-{\eta }_{1}\overline{T }\left(y,s\right),$$

17 $$\frac{{\partial \overline{T}\left( {y,s} \right)}}{\partial y}_{y = 0} = - \left( {\overline{T}\left( {0,s} \right) + \frac{1}{s}} \right),\quad and\quad \overline{T}\left( {y,s} \right) \to 0,\;as\;y \to \infty ,$$

The solution of the Eq. (16) with the boundary condition is in (17), then the transformed solution becomes as under,

18 $$\overline{T} = \frac{1}{{\sqrt {{\text{Pr}}\left( {\frac{{a_{0} s}}{{s + \alpha a_{0} }} + \eta_{1} } \right)} - 1}} \frac{1}{s} exp^{{ - y\sqrt {{\text{Pr}}\left( {\frac{{a_{0} s}}{{s + \alpha a_{0} }} + \eta_{1} } \right)} }} .$$

Using appendices (A_1) and (A_2) we get

$$T\left(y,t\right)=\varphi \left(y,t,{Pra}_{0},Pr{\eta }_{1},\alpha {a}_{0}\right)*\psi \left(y,t,{Pra}_{0},Pr{\eta }_{1},\alpha {a}_{0}\right).$$

### Solution for the velocity equation

By using the Laplace transform method to Eq. (9) and keeping in mind the appropriate initial and boundary condition in (12),

19 $$\frac{{a}_{0}s\overline{u }\left(y,s\right)}{\left(s+\alpha {a}_{0}\right)}=\frac{{\partial }^{2}\overline{u }\left(y,s\right)}{\partial {y}^{2}}+\left(\frac{{a}_{0}\gamma s}{s+\alpha {a}_{0}}\right)\frac{{\partial }^{2}\overline{u }\left(y,s\right)}{\partial {y}^{2}}-M\overline{u }\left(y,s\right)+Gr\overline{T }\left(y,s\right)$$

20 $$\begin{aligned} & \frac{{\partial^{2} \overline{u}\left( {y,s} \right)}}{{\partial y^{2} }} - \left[ {\frac{{a_{0} s + M\left( {s + \alpha a_{0} } \right)}}{{\left( {1 + a_{0} \gamma } \right)s + \alpha a_{0} }}} \right]\overline{u}\left( {y,s} \right) \\ & \quad = \frac{{ - Gr\left( {s + \alpha a_{0} } \right)}}{{\left( {1 + a_{0} \gamma } \right)s + \alpha a_{0} }}\left[ {\frac{1}{{\sqrt {{\text{Pr}}\left( {\frac{{a_{0} s}}{{s + \alpha a_{0} }} + \eta_{1} } \right)} - 1}} \frac{{exp^{{ - y\sqrt {{\text{Pr}}\left( {\frac{{a_{0} s}}{{s + \alpha a_{0} }} + \eta_{1} } \right)} }} }}{s} } \right] \\ \end{aligned}$$

where $a_{0} = \frac{1}{1 - \alpha }$ and $\overline{u}\left( {y,s} \right)$ is a Laplace transform of $u \left( {y,t} \right)$ which has to fulfill the conditions

21 $$\overline{u}\left( {y,s} \right) = \frac{s}{{s^{2} + \omega^{2} }},\quad \overline{u}\left( {y,s} \right) \to 0\quad as\quad y \to \infty$$

By using the above conditions in Eq. (21), the general solution of the Eq. (20) in simpler form is

22 $$\begin{aligned} \overline{u}\left( {y,s} \right) & = \left[ {\frac{s}{{s^{2} + \omega^{2} }} - \left( {\frac{{g_{1} }}{s} + \frac{{g_{2} }}{{s + d_{1} }} + \frac{{g_{3} }}{{s + d_{2} }} + \frac{{g_{4} }}{{s + m_{0} }}} \right)\frac{1}{{\sqrt {{\text{Pr}}\left( {\frac{{a_{0} s}}{{s + \alpha a_{0} }} + \eta_{1} } \right)} - 1}}} \right]e^{{ - y\sqrt {\frac{{b_{1} s + a_{1} }}{{s + a_{2} }}} }} \\ & \quad + \left( {\frac{{g_{1} }}{s} + \frac{{g_{2} }}{{s + d_{1} }} + \frac{{g_{3} }}{{s + d_{2} }} + \frac{{g_{4} }}{{s + m_{0} }}} \right)\frac{{e^{{ - y\sqrt {\frac{{h_{1} s + h_{2} }}{{s + h_{3} }}} }} }}{{\sqrt {{\text{Pr}}\left( {\frac{{a_{0} s}}{{s + \alpha a_{0} }} + \eta_{1} } \right)} - 1}} \\ \end{aligned}$$

Or

23 $$\begin{aligned} \overline{u}\left( {y,s} \right) & = \frac{s}{{s^{2} + \omega^{2} }}e^{{ - y\sqrt {\frac{{b_{1} s + a_{1} }}{{s + a_{2} }}} }} - \frac{{g_{1} }}{s}\frac{{e^{{ - y\sqrt {\frac{{b_{1} s + a_{1} }}{{s + a_{2} }}} }} }}{{\sqrt {\Pr \left( {\frac{{a_{0} s}}{{s + \alpha a_{0} }} + \eta_{1} } \right)} - 1}} - \frac{{g_{2} }}{{s + d_{1} }}\frac{{e^{{ - y\sqrt {\frac{{b_{1} s + a_{1} }}{{s + a_{2} }}} }} }}{{\sqrt {\Pr \left( {\frac{{a_{0} s}}{{s + \alpha a_{0} }} + \eta_{1} } \right)} - 1}} \\ & \quad - \frac{{g_{3} }}{{s + d_{2} }}\frac{{e^{{ - y\sqrt {\frac{{b_{1} s + a_{1} }}{{s + a_{2} }}} }} }}{{\sqrt {\Pr \left( {\frac{{a_{0} s}}{{s + \alpha a_{0} }} + \eta_{1} } \right)} - 1}} - \frac{{g_{4} }}{{s + m_{0} }}\frac{{e^{{ - y\sqrt {\frac{{b_{1} s + a_{1} }}{{s + a_{2} }}} }} }}{{\sqrt {\Pr \left( {\frac{{a_{0} s}}{{s + \alpha a_{0} }} + \eta_{1} } \right)} - 1}} + \frac{{g_{1} }}{s}\frac{{e^{{ - y\sqrt {\frac{{h_{1} s + h_{2} }}{{s + h_{3} }}} }} }}{{\sqrt {\Pr \left( {\frac{{a_{0} s}}{{s + \alpha a_{0} }} + \eta_{1} } \right)} - 1}} \\ & \quad + \frac{{g_{2} }}{{s + d_{1} }}\frac{{e^{{ - y\sqrt {\frac{{h_{1} s + h_{2} }}{{s + h_{3} }}} }} }}{{\sqrt {\Pr \left( {\frac{{a_{0} s}}{{s + \alpha a_{0} }} + \eta_{1} } \right)} - 1}} + \frac{{g_{3} }}{{s + d_{2} }}\frac{{e^{{ - y\sqrt {\frac{{h_{1} s + h_{2} }}{{s + h_{3} }}} }} }}{{\sqrt {\Pr \left( {\frac{{a_{0} s}}{{s + \alpha a_{0} }} + \eta_{1} } \right)} - 1}} + \frac{{g_{4} }}{{s + m_{0} }}\frac{{e^{{ - y\sqrt {\frac{{h_{1} s + h_{2} }}{{s + h_{3} }}} }} }}{{\sqrt {\Pr \left( {\frac{{a_{0} s}}{{s + \alpha a_{0} }} + \eta_{1} } \right)} - 1}} \\ \end{aligned}$$

where

$${b}_{1}=\frac{{a}_{0}+M}{\left(1+{a}_{0}\gamma \right) }, {a}_{1}=\frac{{M\alpha a}_{0}}{(1+{a}_{0}\gamma )}, {a}_{2}=\frac{{\alpha a}_{0}}{(1+{a}_{0}\gamma )}$$

$${m}_{0}=\frac{{\alpha a}_{0}}{1+{a}_{0}\gamma } , {m}_{1}=\frac{-Gr}{(1+{a}_{0}\gamma )(Pr{a}_{0}+Pr{\eta }_{1}-{b}_{1})} ,$$

$${m}_{2}=\frac{Pr{\eta }_{1}{\alpha a}_{0}+Pr{\alpha a}_{2}+Pr{\eta }_{1}{a}_{2}-{a}_{1}-{b}_{1}{\alpha a}_{0}}{Pr{a}_{0}+Pr{\eta }_{1}-{b}_{1}}, {m}_{3}=\frac{Pr{\eta }_{1}{\alpha a}_{0}{a}_{2}-{\alpha a}_{0}{a}_{1}}{Pr{a}_{0}+Pr{\eta }_{1}-{b}_{1}}$$

$${d}_{1}=\frac{{m}_{2}}{2}+\sqrt{{\left(\frac{{m}_{2}}{2}\right)}^{2}+{m}_{3}}$$, $${d}_{2}=\frac{{m}_{2}}{2}-\sqrt{{\left(\frac{{m}_{2}}{2}\right)}^{2}+{m}_{3}}$$

$${g}_{1}=\frac{{m}_{1}{\left({\alpha a}_{0}\right)}^{2}{a}_{2}}{{ d}_{1}{d}_{2}{m}_{0}}$$, $${g}_{2}=\frac{{m}_{1}{\left(-{ d}_{1}+{\alpha a}_{0}\right)}^{2}(-{ d}_{1}+{a}_{2})}{{-d}_{1}({- d}_{1}+{d}_{2})(-{ d}_{1}+{m}_{0})},$$ $${g}_{3}=\frac{{m}_{1}{\left(-{ d}_{2}+{\alpha a}_{0}\right)}^{2}(-{ d}_{2}+{a}_{2})}{{-d}_{2}({- d}_{2}+{d}_{1})(-{ d}_{2}+{m}_{0})} ,{g}_{4}=\frac{{m}_{1}{\left(-{ m}_{0}+{\alpha a}_{0}\right)}^{2}(-{ m}_{0}+{a}_{2})}{-{ m}_{0}({- m}_{0}+{d}_{1})(-{m}_{0}+{ d}_{2})}$$

$${h}_{1}=\mathrm{Pr}\left({a}_{0}+{\eta }_{1}\right), {h}_{2}=Pr{\eta }_{1}\alpha {a}_{0}, {h}_{3}=\alpha {a}_{0}$$

Using appendix (A_1) and (A_3) we get

$$\begin{aligned} u\left( {y,t} \right) & = \left[ {\cos \omega t - \left( {g_{1} + g_{2} e^{{ - d_{1} t}} + g_{3} e^{{ - d_{2} t}} + g_{4} e^{{ - m_{0} t}} } \right)*\varphi \left( {y,t,Pra_{0} ,Pr\eta_{1} ,\alpha a_{0} } \right)} \right] \\ & \quad *\phi \left( {y,t,b_{1} ,a_{1} ,a_{2} } \right) + \left( {g_{1} + g_{2} e^{{ - d_{1} t}} + g_{3} e^{{ - d_{2} t}} + g_{4} e^{{ - m_{0} t}} } \right) \\ & \quad *\varphi \left( {y,t,Pra_{0} ,Pr\eta_{1} ,\alpha a_{0} } \right)*\phi \left( {y,t,h_{1} ,h_{2} ,h_{3} } \right) \\ \end{aligned}$$

For Special Case $\alpha =1$:

When $$\alpha = 1$$ then $$a_{0} = \frac{1}{1 - \alpha }$$ $$\to \infty$$

$$b_{1} = \frac{1}{\gamma },\quad a_{1} = \frac{M}{\gamma },\quad a_{2} = \frac{1}{\gamma }$$

$$m_{0} = \frac{1}{\gamma },\quad m_{1} = 0,$$

$$m_{2} = \frac{{Pr\eta_{1} + Pr - b_{1} }}{Pr},\quad m_{3} = \frac{{Pr\eta_{1} a_{2} - a_{1} }}{Pr}$$

$$d_{1} = \frac{{m_{2} }}{2} + \sqrt {\left( {\frac{{m_{2} }}{2}} \right)^{2} + m_{3} } ,\quad d_{2} = \frac{{m_{2} }}{2} - \sqrt {\left( {\frac{{m_{2} }}{2}} \right)^{2} + m_{3} }$$

$$g_{1} = 0$$, $$g_{2} = 0,$$ $$g_{3} = 0 ,g_{4} = 0$$ because m_1_ = 0

$$h_{1} = \infty ,\quad h_{2} = \infty ,\quad h_{3} = \infty$$

$$u\left( {y,t} \right) = \left[ {\cos \omega t*\varphi \left( {y,t, b_{1} ,a_{1} ,a_{2} } \right)} \right]$$

$$\begin{aligned} \vartheta \left( {y,t,a_{1} ,b_{1} ,a_{1} b_{2} ,} \right) & = L^{ - 1} \left[ {\exp \left( { - y\sqrt {\frac{{a_{1} s + b_{1} }}{{s + b_{2} }}} } \right)} \right] \, = \delta \left( t \right)e^{{ - y\sqrt {a_{1} } }} + \int_{0}^{\infty } {\frac{y}{2u\sqrt \pi }\sqrt {\frac{{a_{1} b_{2} - b_{2} }}{t}} } \\ & \quad \times e^{{\frac{{ - y^{2} }}{4u}}} \times e^{{ - b_{2} t - a_{1} u}} \, \times I_{1} \left( {2\sqrt {\left( {a_{1} b_{2} - b_{2} } \right)ut} } \right)du. \\ \end{aligned}$$

*Special Cases*

(i) In the absence of heat generation $({\eta }_{1}=0)$ and considering T(0,t) = T_w_ condition of fluid temperature in place of ${\frac{\partial T\left(y,t\right)}{\partial y}|}_{y=0}=-\left(T\left(0,t\right)+1\right) we get;$
  The result given (24) is uniform to the published literature given in ^16^ .
  24 $$\begin{aligned} & T(y,t) = \varphi (y,t;\Pr \gamma ,\alpha \gamma )\quad 0 < \alpha < 1 \\ & {\text{where }} \\ & \varphi (y,t;\Pr \gamma ,\alpha \gamma ) = 1 - \frac{2\Pr \gamma }{\pi }\mathop \smallint \limits_{0}^{\infty } \frac{{\sin \left( {yx} \right)}}{{x\left( {\Pr \gamma + x^{2} } \right)}}\exp \left( {\frac{{ - \alpha \gamma tx^{2} }}{{\Pr \gamma + x^{2} }}} \right)dx, \\ \end{aligned}$$
(ii) In the absence of MHD effect and considering $u(0,t) = fH(t)e^{i\omega t}$ condition of fluid’s velocity at boundary in place of $u\left(0,t\right)=H(t)\mathrm{cos}\omega t$ condition we get;
  25 $$\begin{aligned} u(y,t)& = U_{1} (y,t) + U_{2} (y,t) + \psi (y,t;a_{1} ,a_{2} ,i\omega ) \\ & {\text{where}} \\ & U_{1} (y,t) = (1 - d_{1} - d_{3} )\varphi (y,t;a_{1} ,a_{2} ) + (d_{1} + d_{3} )\varphi (y,t;\Pr \gamma ,\alpha \gamma ) \\ & \quad + d_{3} [\psi (y,t;\Pr \gamma ,\alpha \gamma , - b_{2} ) - \psi (y,t;a_{1} ,a_{2} , - b_{2} )] \\ & U_{2} (y,t) = d_{2} \int\limits_{0}^{t} {\left[ {\varphi (y,t;\Pr \gamma ,\alpha \gamma ) - \varphi (y,t;a_{1} ,a_{2} )} \right]} d\tau \\ \end{aligned}$$

The result given (25) is uniform to the published literature given in^16^.

## Numerical results and discussion

**Figure 2:**
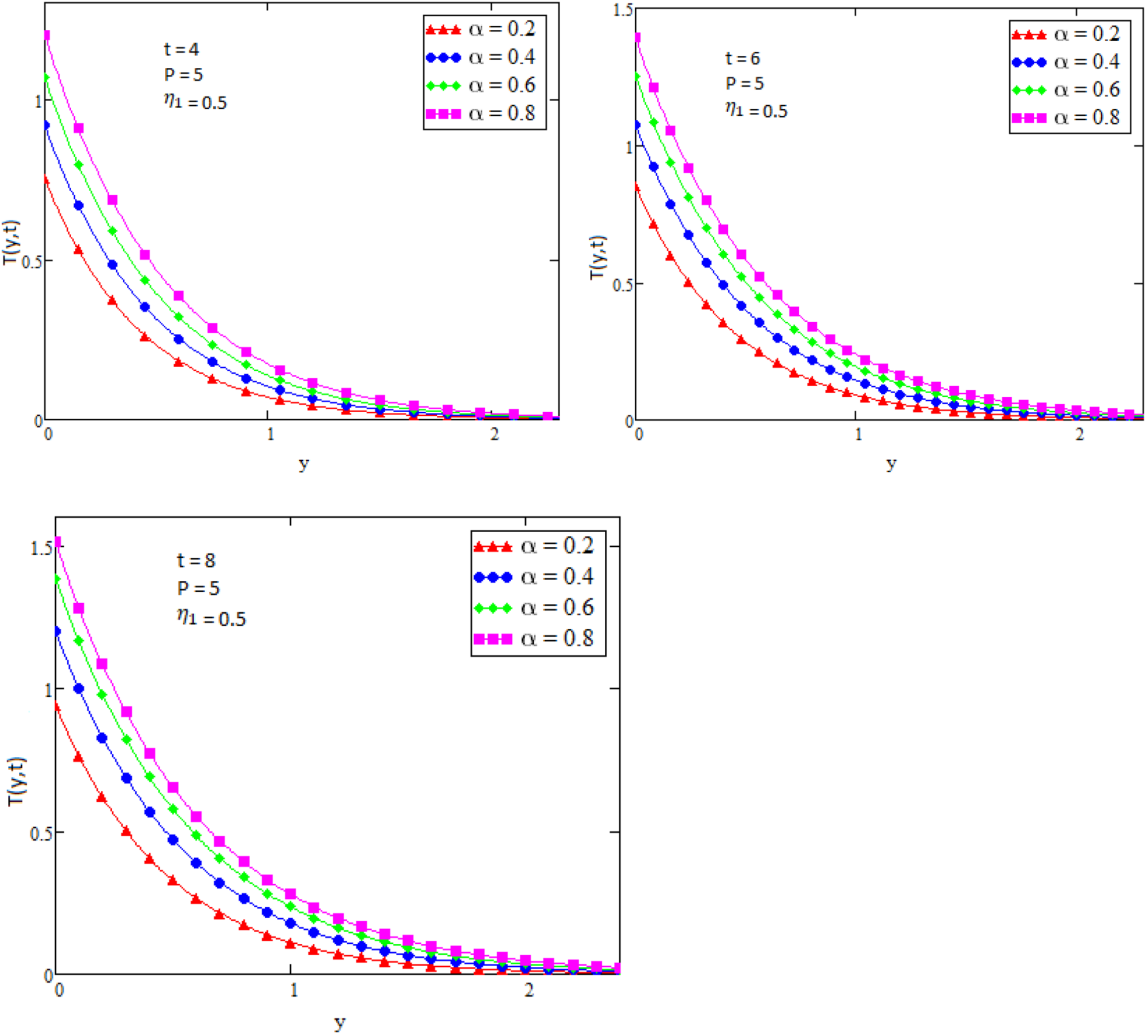
Temperature profile for different values of Alpha $\alpha$.

**Figure 3:**
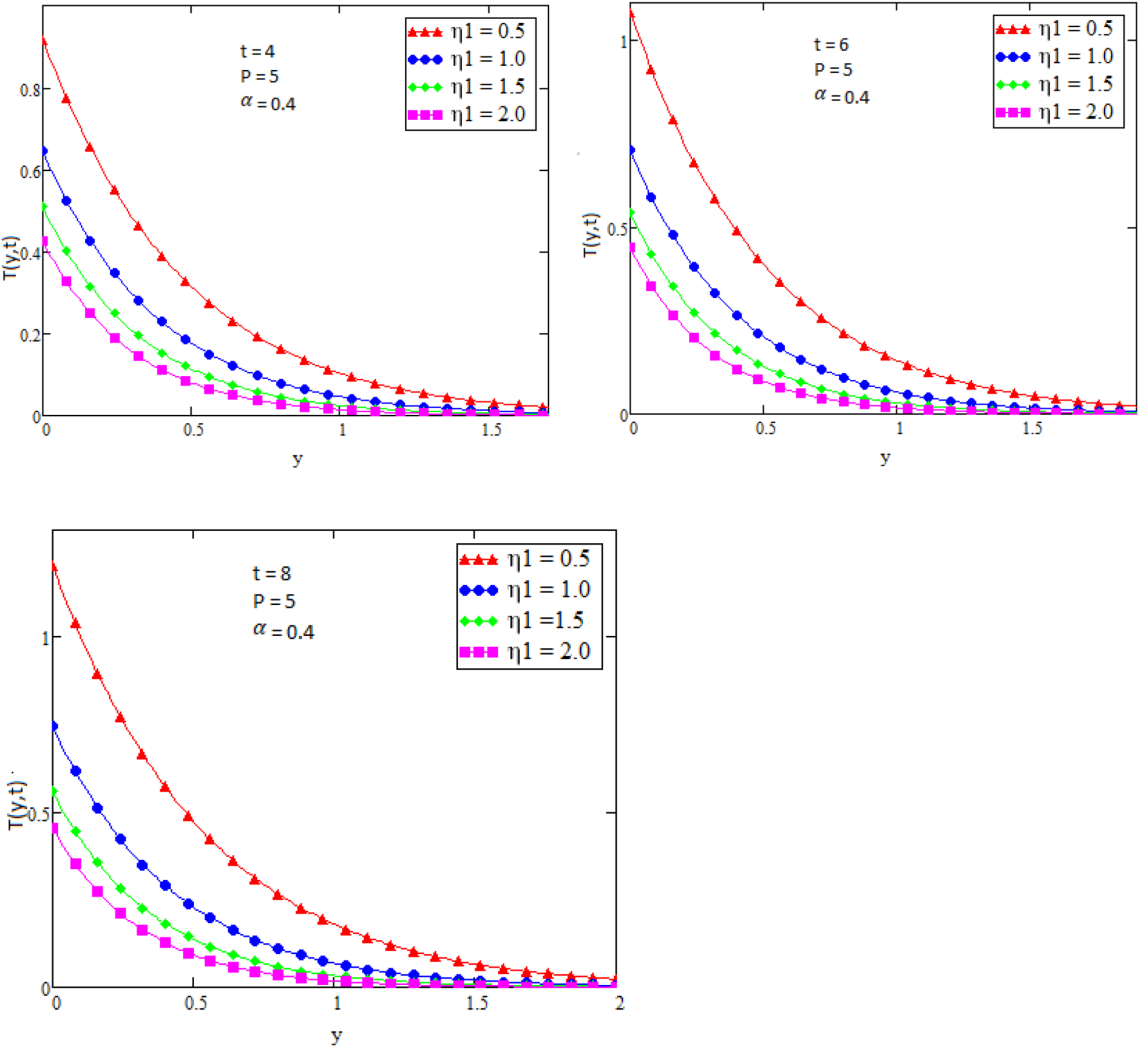
Temperature profile for different values of Eta ${\eta }_{1}$.

**Figure 4:**
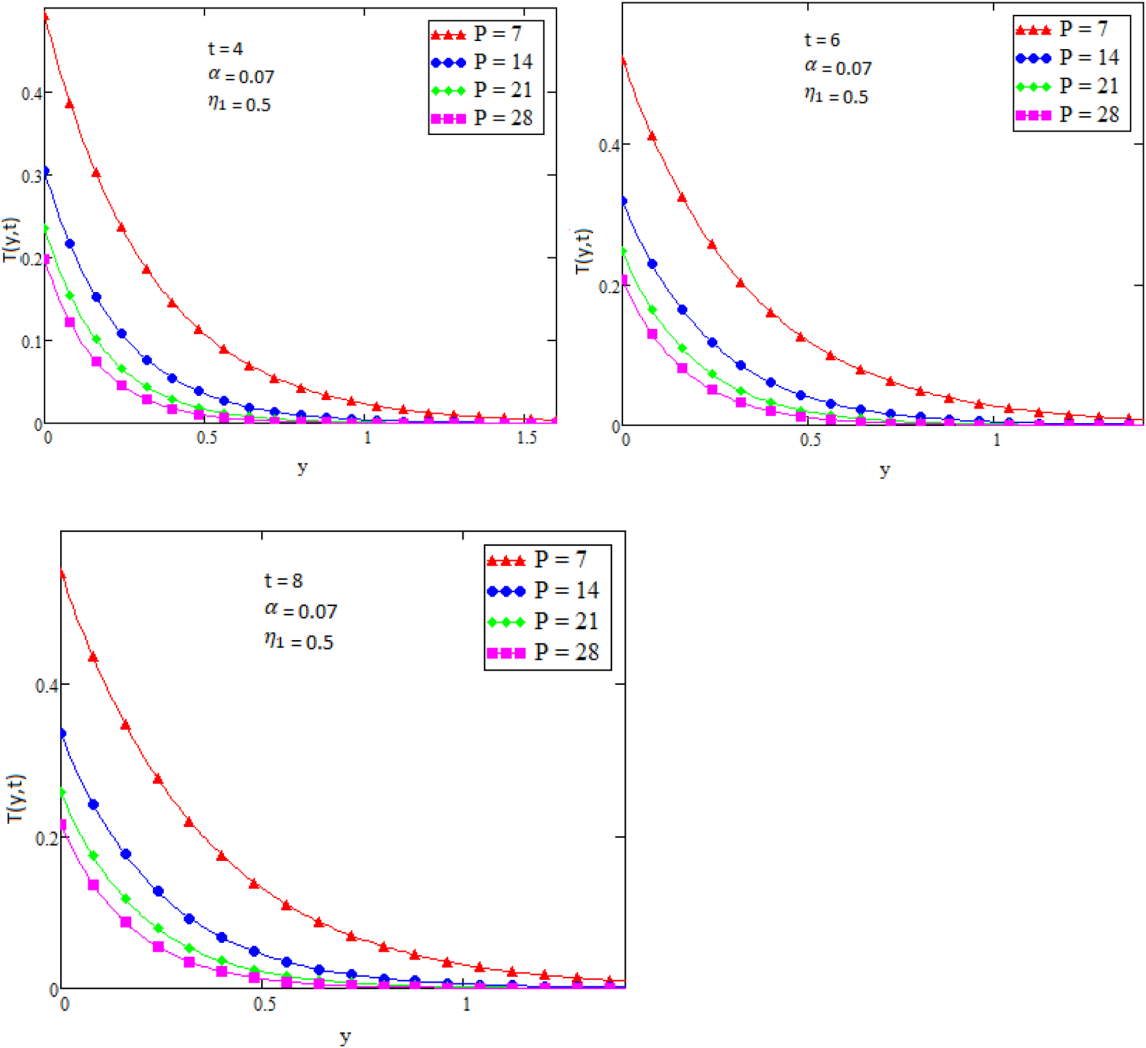
Temperature profile for different values of Prandtl number Pr.

**Figure 5:**
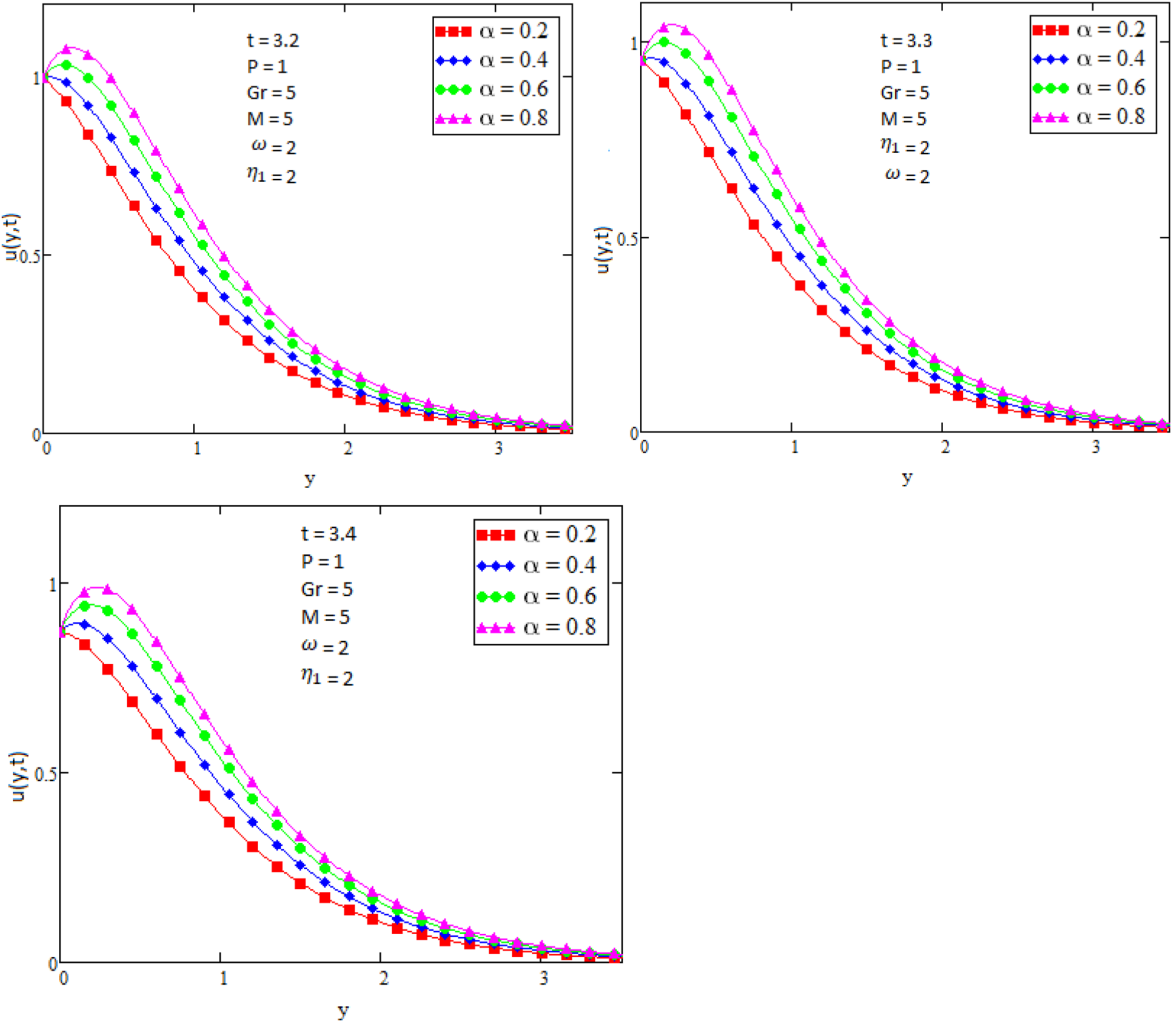
Velocity profile for different values of Alpha $\alpha$.

**Figure 6:**
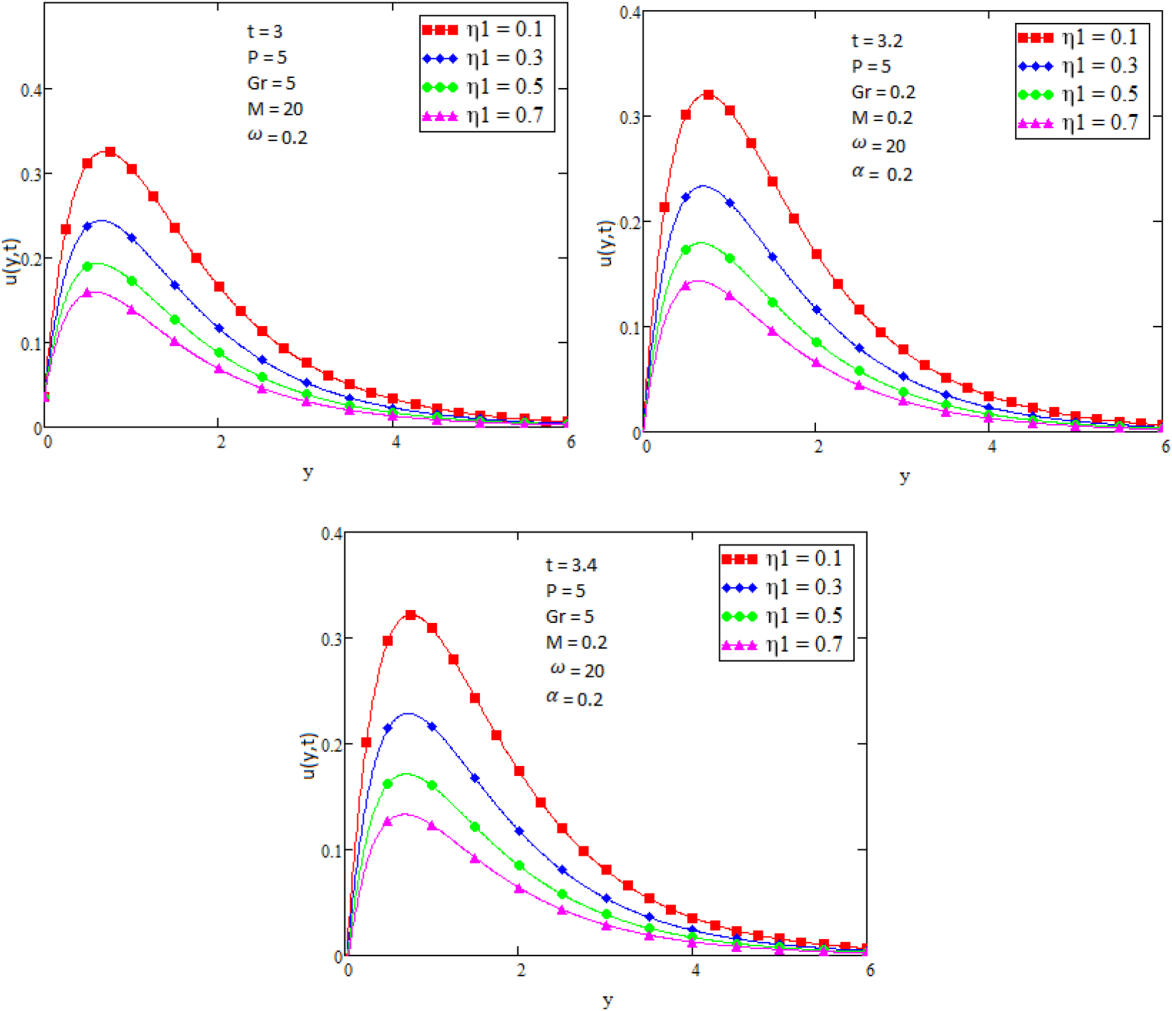
Velocity profile for different values of Eta ${\eta }_{1}$.

**Figure 7:**
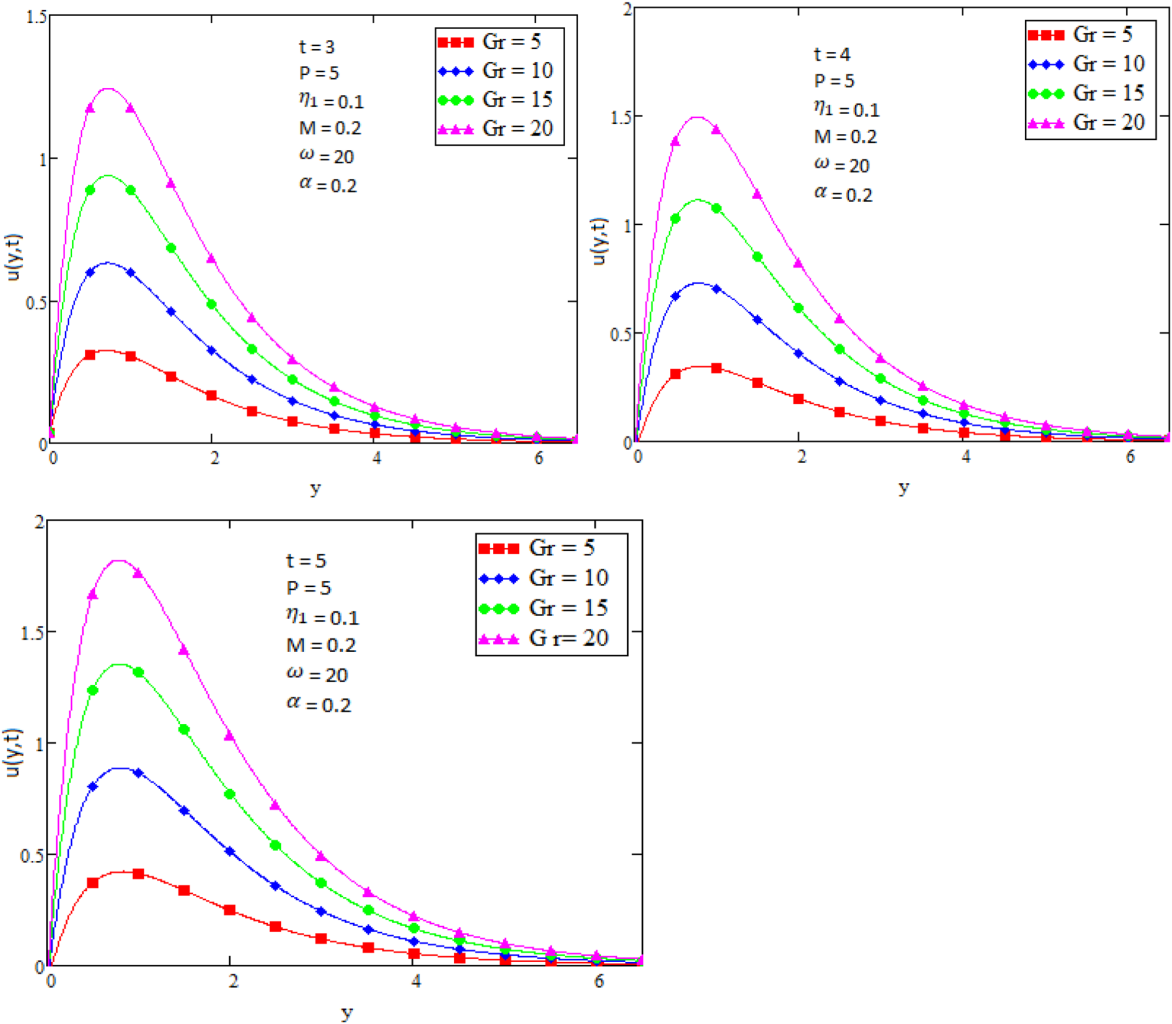
Velocity profile for different values of Grashof number Gr.

**Figure 8:**
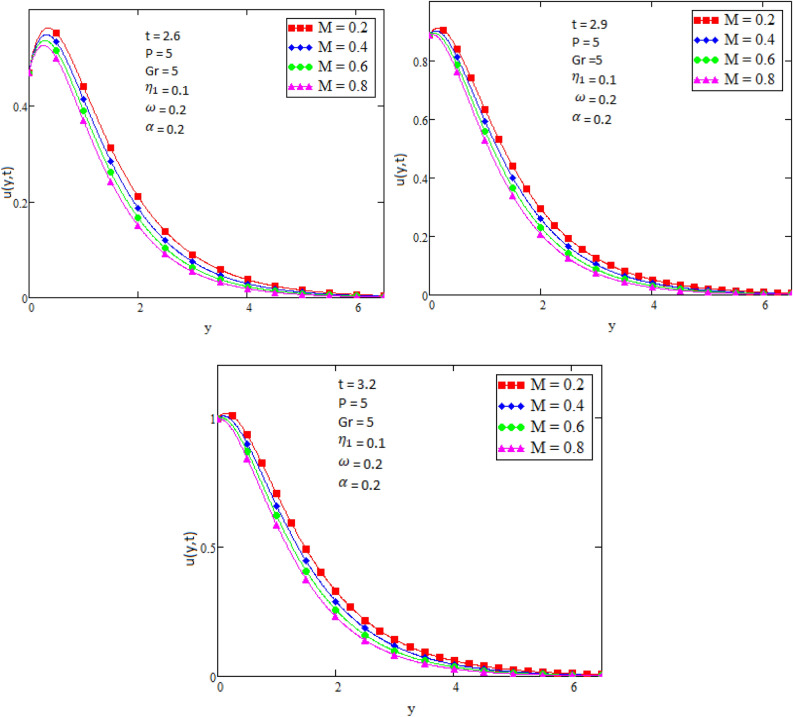
Velocity profile for different values of MHD.

**Figure 9:**
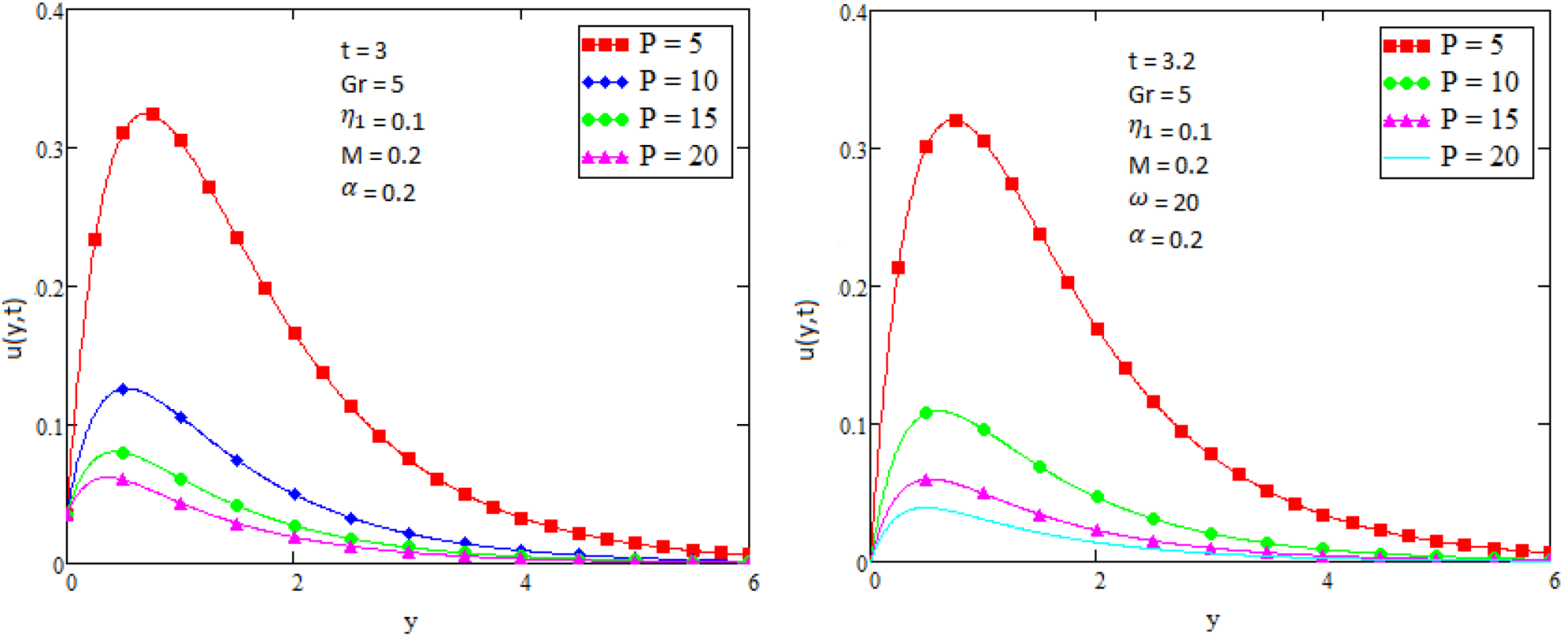
Velocity profile for different values of Prandtl number Pr.

**Figure 10:**
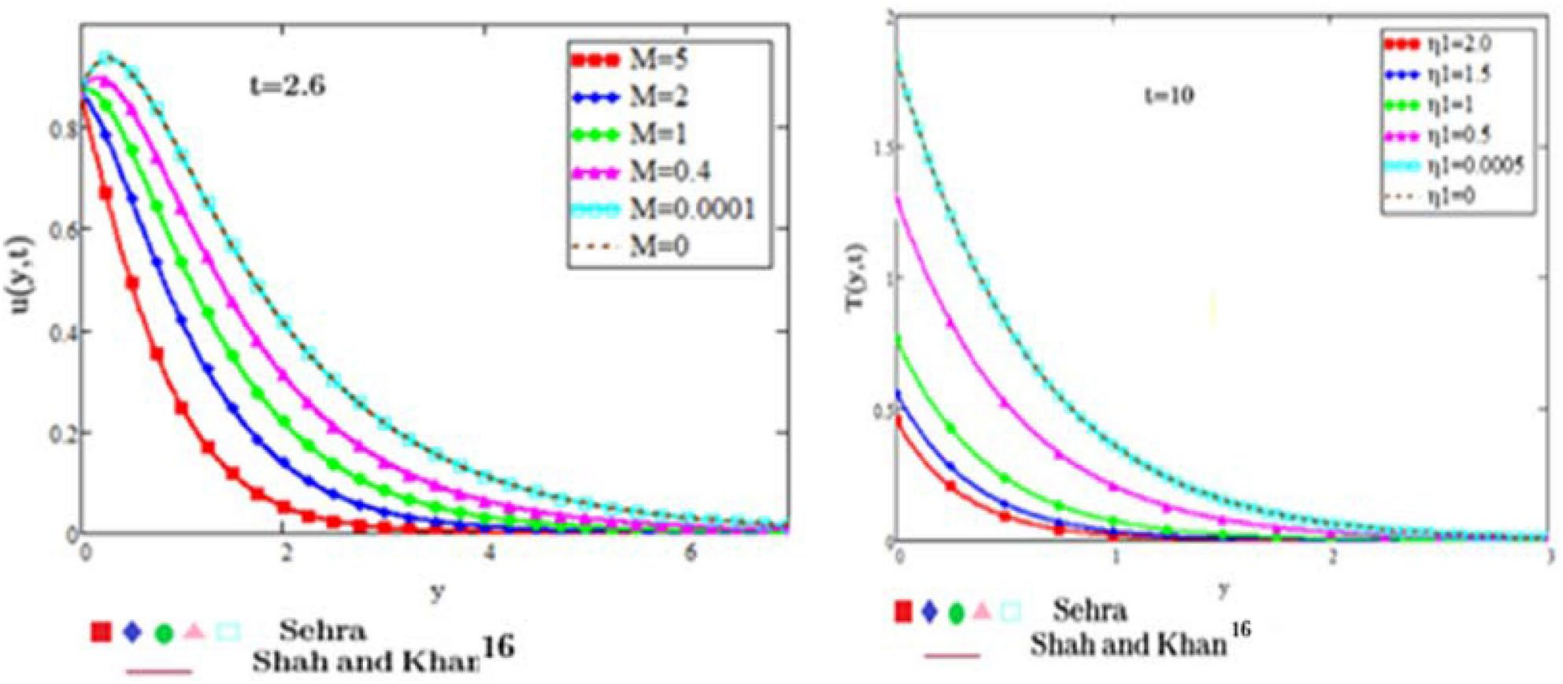
Velocity and temperature profiles in comparison to the results in^16^.

**Figure 11:**
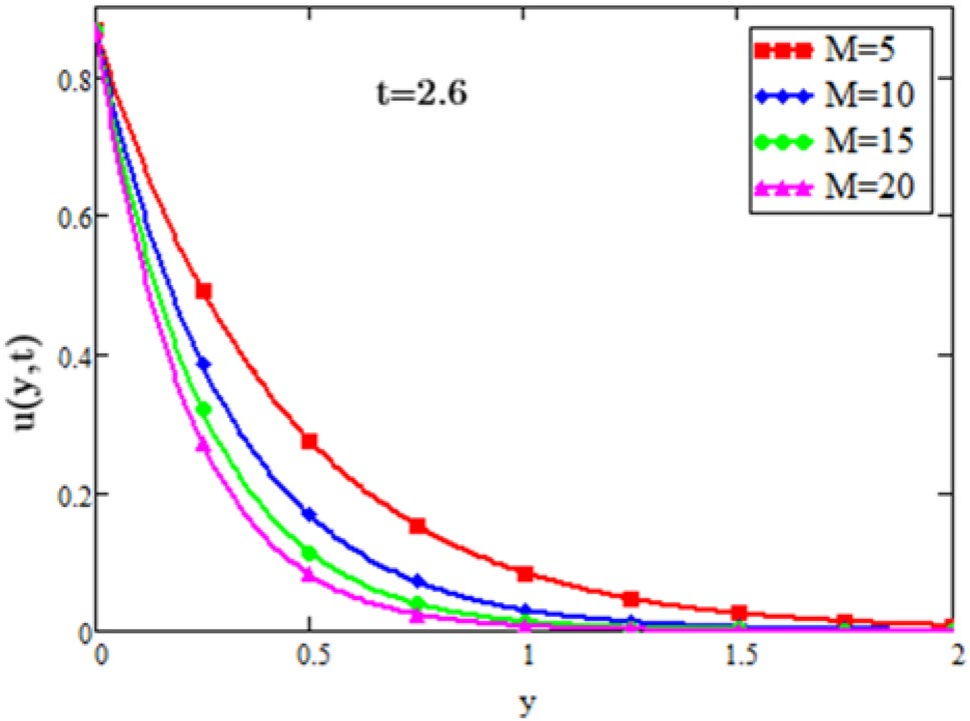
Velocity profiles for special case alpha = 1.

By using Mathcad software different physical parameters were drawn to analyze the effects of fluid velocity and temperature. The parameter Alpha $\alpha$ in Fig. 2, Eta ${\eta }_{1}$ in Fig. 3, and Prandtl number Pr in Fig. 4 are sketched for temperature field, while for velocity field the Alpha $\alpha$ in Fig. 5, Eta ${\eta }_{1}$ in Fig. 6, Grashof number Gr in Fig. 7, Magneto hydrodynamic MHD in Fig. 8 and Prandtl number Pr in Fig. 9 are presented with different values of time t. Figure 2 is drawn to show the effects of alpha $\alpha$ for temperature profile in which it is observed that by increasing the value of alpha $\alpha ,$ the temperature is also increases. In this way the consistency of thermal boundary layer is increases with the parameter alpha $\alpha$ and time t. Figure 3 is sketched to check the effect eta ${\eta }_{1}$ for temperature profile in which it is observed that by increasing the value of eta ${\eta }_{1}$ the temperature decreases, the consistency of thermal boundary layer also decreases with the parameter eta ${\eta }_{1}$ and time t. Figure 4 is sketched to examine the effects of the Prandtl number Pr in which it is noticed that by increasing the values of the parameter Pr, the temperature profile decreases, as Prandtl number is the ratio of momentum diffusivity to thermal conductivity by increasing the Prandtl number thermal conductivity decreases which cause to decrease the temperature of the fluid. The Fig. 5 is drawn to examine the effects of fractional parameter alpha $\alpha$ on the velocity profile of the fluid, and it is concluded that the velocity of the fluid increases with increasing values of fractional parameter alpha $\alpha$. Figure 6 is drawn to show the effects of ${\eta }_{1}$ on fluid velocity. From Fig. 6 it is noticed that velocity of the fluid have inverse relation with the parameter eta ${\eta }_{1}$, the velocity of the fluid decreases with the increasing values of ${\eta }_{1}$. Figure 7 is sketched to examine the effect of Grashof Gr number for velocity profile, here it is noticed that by increasing the value of Grashof number the fluid velocity is increases, because Grashof number is the ratio of inertia to viscous force, Grashof number is inversely proportional to viscous force, so increase in Grashof number cause decrease in viscosity. It is obvious that for low viscosity the velocity is higher. That is why for increasing values of Grashof number the fluid velocity increases. Figure 8 is drawn to show the effects of magneto hydrodynamic MHD on fluid velocity, here it is noticed that by increasing the value of Magneto hydrodynamic the motion of fluid is decreases. Figure 9 are sketched to examine the effect of Prandtl number Pr on velocity fluid, where we noticed that by increasing the value of Prandtl number, the velocity of the fluid is decreases. Figure 10 is shown in comparison with published result obtained in^16^. Figure 11 is shown for $\alpha$=1. From obtained results by neglecting magneto hydrodynamic and heat source some special case are achieved which are published in literature published by Shah and Khan in^16^. The case for which the fractional parameter approaches to the classical order also discussed and it has been observed that it is convergent.

## Conclusion

The considered study is about analyze the unsteady natural convection flow of generalized second grade fluid with magneto hydro dynamic effects and Newtonian heating in addition to heat generation. Some special cases of the obtained solution are discussed from which some well-known results are found in the published literature which are similar to published in^16^. The exact solution of the problem achieved by using Laplace transform technique are represented graphically to visualize the effects of physical parameters like time fractional, Magneto hydrodynamic, Prandtl number, heat source and Grashof Number. From graphical results it is concluded that;

- The increasing values of the Prandtl number and heat source reduce the temperature of the fluid.
- The increasing values of the fractional parameter alpha increases the temperature of the fluid.
- The increasing values of Prandtl number, heat source and MHD reduces the velocity of the fluid.
- The increasing values of the fractional parameter alpha and Grashof number increases the velocity of the fluid.
- By neglecting the heat source temperature equation and MHD in velocity equation we obtained a published which is given in^16^.

## Supplementary Information

Supplementary Information.

## Data Availability

The datasets used and analyzed during the current study available from the corresponding author on reasonable request.

## List of symbols

u [*LT*^−1^]: Fluid velocity
T [*θ*]: Temperature
t [*T*]: Time
C_*p*_ [*L*^2^*MT*^−1^*θ*^−1^]: At constant pressure the specific heat
g [*LT*^−2^]: Acceleration due to gravity
k [*MLT*^−3^*θ*^−1^]: Thermal conductivity or heat conduction of the fluid
*µ* [*ML*^−1^*T*^−1^]: Dynamic viscosity
*ν* [*L*^2^*T*^−1^]: Kinematic viscosity
*ρ* [*ML*^−3^]: Fluid’s density
*β*_*T*_ [*θ*^−1^]: Coefficient of volumetric expansion of thermal
T_*w*_ [*θ*]: At plate the fluid temperature
T_∞_ [*θ*]: The fluid temperature away from the plate
Gr: Grashof number of thermal
*α*_1_: Second grade parameter (Dimensional)
*γ*: Second grade parameter (Dimensionaless)
Pr $\left( {\frac{{\mu C_{p} }}{k}} \right)$: Prandtl number
s: Parameter of Laplace transform
