# Supplementary material for: Caputo–Fabrizio fractional model of MHD second grade fluid with Newtonian heating and heat generation
Source: Sci Rep. 2022 Dec 26;12:22371. doi: 10.1038/s41598-022-26080-7 (PMC9792569; doi:10.1038/s41598-022-26080-7)
Supplement: Supplementary file 1 — Supplementary Information. [file 41598_2022_26080_MOESM1_ESM.pdf]

Appendices

$$\varphi(y,t,a.b,c) = \delta(t)\int_0^\infty e^{-ua-b}\left\lfloor \frac{1}{\sqrt{\pi t}} + e^{u-b}\left(2 - erfc\left(\sqrt{u}\right)\right)\right\rfloor du + \int_0^\infty \left\lfloor \frac{1}{\sqrt{\pi t}} + e^{u-b}\left(2 - erfc\left(\sqrt{u}\right)\right)\right\rfloor \sqrt{\frac{uac}{t}}\, e^{-u\alpha_1 Pr-b-ct} I_0\left(2\sqrt{uact}\right) du \quad \text{(A\_1)}$$

$$\psi(y,t,a,b,c) = 1 - \frac{2a}{\pi}\int_0^\infty \frac{\sin(yu)}{u(u^2+a)} exp\left(\frac{-ctu^2-b}{u^2+a}\right) du \quad \text{(A\_2)}$$

$$\phi(y,t,m_1,m_2,m_3) = L^{-1}\left\lfloor exp\left(-y\sqrt{\frac{m_1 s+m_2}{s+m_3}}\right)\right\rfloor = \delta(t)e^{-y\sqrt{m_1}} + \int_0^\infty \frac{y}{2u\sqrt{\pi}}\sqrt{\frac{m_1 m_3 - m_2}{t}} \times e^{\frac{y^2}{4u}} \times e^{-m_3 t - m_1 u} \times I_1\left(2\sqrt{(m_1 m_3 - m_2)ut}\right) du. \quad \text{(A\_3)}$$
